# Process evaluation of a tailored intervention programme of cardiovascular risk management in general practices

**DOI:** 10.1186/s13012-016-0526-z

**Published:** 2016-12-15

**Authors:** E. Huntink, M. Wensing, I. M. Timmers, J. van Lieshout

**Affiliations:** 1 Department IQ healthcare, Radboud Institute of Health Sciences, Radboud university medical center, PO Box 9101, 6500 HB Nijmegen, The Netherlands; 2 Department of General Practice and Health Services Research, Heidelberg University Hospital, Marsilius Arkaden, Turm West, Im Neuenheimer Feld 130.3, 69120 Heidelberg, Germany

**Keywords:** Process evaluation, Practice nurses, Cardiovascular disease, Tailored implementation programme, Depressive symptoms, Motivational interviewing, CVRM knowledge, CVRM patients

## Abstract

**Background:**

A tailored implementation programme to improve cardiovascular risk management (CVRM) in general practice had little impact on outcomes. The questions in this process evaluation concerned (1) impact on counselling skills and CVRM knowledge of practice nurses, (2) their use of the various components of the intervention programme and adoption of recommended practices and (3) patients’ perceptions of counselling for CVRM.

**Methods:**

A mixed-methods process evaluation was conducted. We assessed practice nurses’ motivational interviewing skills on audio-taped consultations using Motivational Interviewing Treatment Integrity (MITI). They also completed a clinical knowledge test. Both practice nurses and patients reported on their experiences in a written questionnaire and interviews. A multilevel regression analysis and an independent sample *t* test were used to examine motivational interviewing skills and CVRM knowledge. Framework analysis was applied to analyse qualitative data.

**Results:**

Data from 34 general practices were available, 19 intervention practices and 14 control practices. No improvements were measured on motivational interviewing skills in both groups. There appeared to be better knowledge of CVRM in the control group. On average half of the practice nurses indicated that they adopted the recommended interventions, but stated that they did not necessarily record this in patients’ medical files. The tailored programme was perceived as too large. Time, follow-up support and reminders were felt to be lacking. About 20% of patients in the intervention group visited the general practice during the intervention period, yet only a small number of these patients were referred to recommended options.

**Conclusions:**

The tailored programme was only partly used by practice nurses and had little impact on either their clinical knowledge and communication skills or on patient reported healthcare. If the assumed logical model of change is valid, a more intensive programme is needed to have an impact on CVRM in general practice at all.

## Background

In the Netherlands, patients with established cardiovascular disease (CVD) or at high cardiovascular risk are mainly treated in general practice [1]. Although many efforts have been made to implement the multidisciplinary practice guideline cardiovascular risk management (CVRM) [2] and the ‘care standard’ [3], which provides clinical and organisational recommendations, not all patients receive recommended preventive interventions [4, 5]. Self-management is a crucial component of CVRM, yet about half of the patients are insufficiently active in self-management due to lack of motivation, knowledge and skills [6].

A programme of tailored interventions, focused on improving and facilitating prospectively identified determinants of current practice, may contribute to the improvement of quality of care [7]. The international project tailored intervention for chronic diseases (TICD) focused on the development and evaluation of a tailored implementation programme [8]. We followed subsequent steps starting with identifying barriers and enablers (so-called determinants) of current practice of CVRM care in general practice in the Netherlands and strategies to address these [9–11]. Next, we developed a tailored intervention programme, comprising of the prioritised strategies. This intervention programme was largely targeted at improving counselling skills of practice nurses treating patients with established CVD or at high cardiovascular risk and their knowledge of CVRM. Patients at high cardiovascular risk have a 10-year risk score of 20% or higher for morbidity and mortality due to CVD [2]. To evaluate the intervention programme, we performed a two arm, cluster randomised trial in seven provinces in the Netherlands in 2013 and 2014. The outcome evaluation showed hardly any improvements in the delivery of CVRM [12]. Parallel to the outcome evaluation, we conducted a process evaluation to explore explanations for the study outcomes. The questions of this process evaluation concerned (1) impact on counselling skills and CVRM knowledge of practice nurses, (2) their use of the various components of the intervention programme and adoption of recommended practices and (3) patients’ perceptions of counselling for CVRM.

## Methods

Details of the study design and methods have been reported elsewhere [13]. In this article, we will focus on the methods relevant for this process evaluation.

### Study design

In 2013–2014, we conducted a cluster randomised trial in the Netherlands to evaluate a tailored implementation programme that formed part of the TICD project. The primary outcome was a record in the patient’s medical file about lifestyle counselling or an appropriate referral for patients with depressive symptoms. The secondary outcomes comprised of cardiovascular risk factors and patient-reported lifestyle behaviours. Data were collected from patients’ medical files and questionnaires aimed at patients [12]. The same group of practice nurses and patients provided us with data for the process evaluation. For this process evaluation, we used a mixed-methods design. Qualitative components involved interviews with practice nurses in the intervention group and a sample of 12 patients with established CVD or at high cardiovascular risk. Quantitative components were questionnaires for all participating practice nurses and patients, and scores of audio-taped interviews of practice nurses before and after the intervention period. In the context of the TICD project, a framework including seven domains was developed to identify determinants of practice [14], which was used to organise our data. Data for this process evaluation were collected between April and September 2014. The ethical committee of Arnhem and Nijmegen waved approval for the study (2013/229).

### Participants

In total 44 practice nurses expressed an interest in participating and gave their written informed consent. Eligible practice nurses treated patients with established CVD or at high cardiovascular risk and had already been trained for motivational interviewing during their vocational training or as part of their continued education. Practice nurses provide patients with lifestyle advice, perform biomedical measurements and consult general practitioners (GPs) about drug treatment. Patients with established CVD or at high cardiovascular risk were selected in cooperation with their practice nurse. Eligible patients met the International Classification of Primary Care (ICPC) codes K74-76, K85-K92, K99.1, and T93; sometimes, two codes or more needed to be present in order to determine high cardiovascular risk, depending on age, gender and smoking status. Patients were 18 years of age or older and had to be able to fill out an informed consent form. Patients were excluded if they had diabetes mellitus, pregnancy or lactation, terminal illness, cognitive impairment and/or poor language skills. We excluded CVRM patients with diabetes mellitus because the results then would have been influenced by the quality of diabetes care with a longer history of programmed care. In this article, we will exclusively focus on CVRM care.

### Tailored implementation programme

**Fig. 1:**
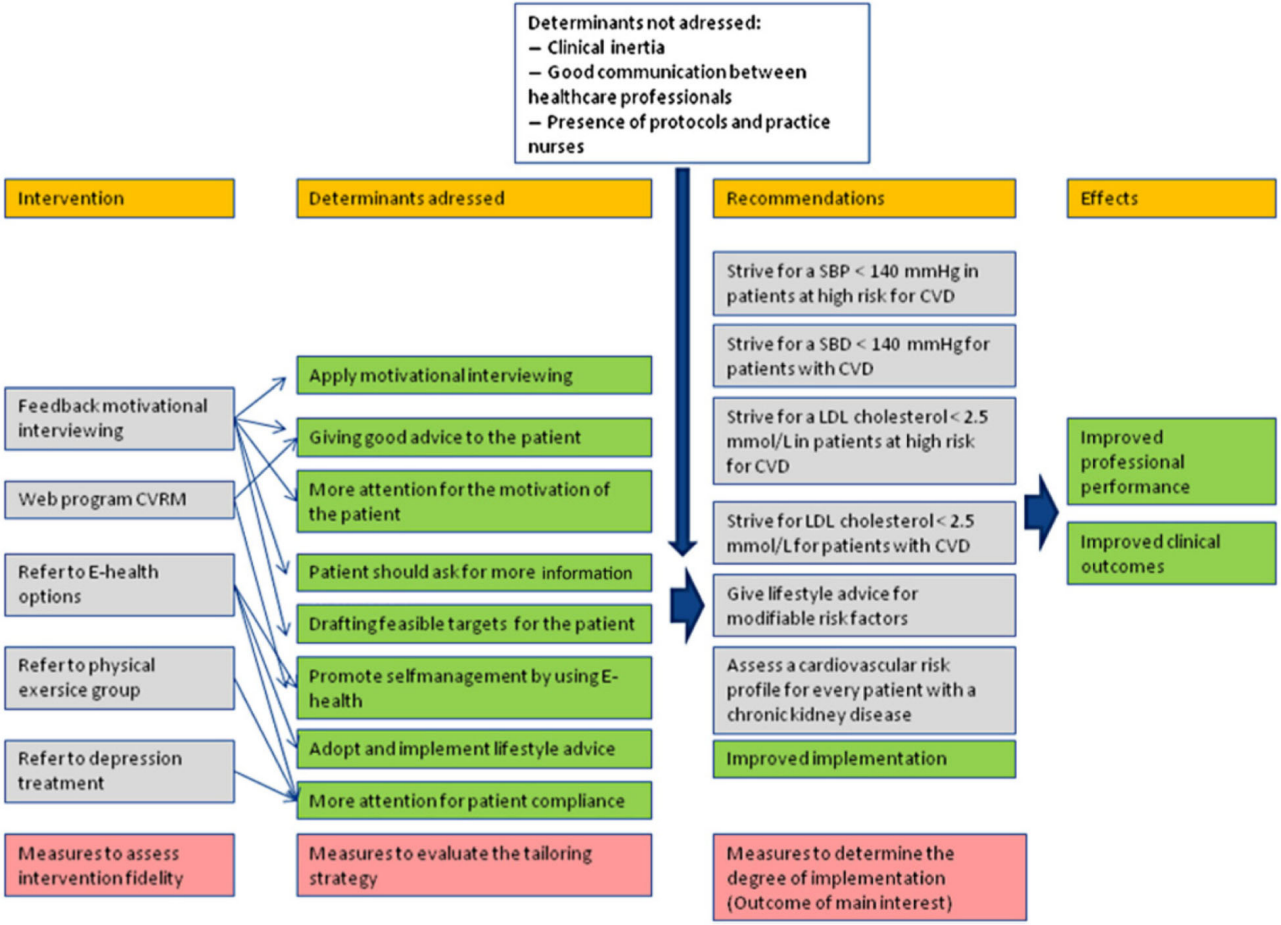
Logic model: This figure provides information regarding which determinants and recommendations are addressed to the intervention program and which are not addressed, as well as showing the intended effect

During the TICD project, we developed a tailored intervention programme by following sequential steps, in order to enhance the quality of CVRM care. First, targets for improvement were determined by analysing clinical guidelines [2, 3] and clinical audit data. Selected targets were systolic blood pressure (SBP) <140 mmHg in patients with established CVD or at high cardiovascular risk; low density lipoprotein (LDL) cholesterol <2.5 mmol/l in patients with established CVD or at high cardiovascular risk; promote lifestyle changes for patients with established CVD or at high cardiovascular risk; create a risk profile for patients with chronic kidney disease. Subsequently, interviews were held with healthcare professionals and patients whereby 139 plausibly important determinants of practice were identified. We selected 11 determinants based on importance and changeability. During group interviews, stakeholders and patients suggested 181 strategies for implementation to influence the selected determinants; these strategies were used for developing the tailored intervention programme considering feasibility and potential impact, see Fig. 1. The intervention programme consisted of the following components: (1) A mandatory feedback training on motivational interviewing for practice nurses to enhance their motivational interviewing skills and to address the determinants of drafting feasible targets for the patients, giving patients good advice and thereby to improve patients’ motivation for a better lifestyle. (2) A new educational web programme (CVRM) was offered to enhance practice nurses’ knowledge about CVRM, and subsequently the determinants of giving patients good advice and improve their self-management. (3) We formulated the recommendation to categorise the patients in three groups based on the presence of depressive symptoms and tailor care accordingly, because patients without, with mild or with severe depressive symptoms benefit from a different approach [15]. The Patients’ Health Questionnaire (PHQ-9) is an instrument for screening, diagnosing, monitoring and measuring the severity of depression, which we offered as supportive material [16]. (4) Patients without depressive symptoms were provided with an information card containing an option to write down personal target values for blood pressure and LDL cholesterol. E-health options were also written down on this information card, namely ‘thuisarts.nl’ and ‘hartenvaatgroep.nl’, as well as Twitter consultation options. Practice nurses were asked to explain the information on the card. The E-health options offered addressed the following determinants: to enhance patients’ self-management by using E-health, to improve patients’ adoption and implementation of lifestyle advice and to enhance patients’ compliance. (5) The recommendation was to refer patients with mild depressive symptoms to a physical exercise group. This could be an exercise group led by a physical therapist or ‘Nederland in beweging’ (‘the Netherlands on the move’), a Dutch television programme. Physical exercise has a beneficial effect on CVD and on depressive symptoms, but we also intended to influence the determinant ‘more attention for patients’ compliance’. For patients with severe depressive symptoms, we advised the practice nurse to refer these patients to their GP, practice nurse mental health or psychologist, as appropriate within the general practice. Severe depressive symptoms negatively influence patients’ compliance [2]; for that reason, we recommended reducing depressive symptoms before starting with lifestyle advice. Practice nurses in the control group were asked to provide their usual care.

Following a published framework [17], we introduced the intervention programme as follows: three members of the TICD project individually visited each participating general practice, which lasted in general 1 h, to instruct practice nurses. They used a standardised script in order to ensure that the intervention programme was clear and that the practice nurses were motivated. Further contacts were related to practical aspects of the feedback training motivational interviewing, the web programme and the handing in of audio-taped patient consultations.

### Data collection

Data was collected at the start and at follow-up, which was planned 6 months after the start, but due to practical constraints, it was actually 7 up to 11 months after delivery of the intervention programme.

#### Quantitative measures

To assess how practice nurses applied their motivational interviewing skills, we asked them to hand in two audio tapes of their consultations with patients, one at baseline before the feedback training and an audio tape at follow-up. The audio tapes were transcribed verbatim. Transcriptions were scored by professional trainers, who were affiliated with MINTned (Association of Dutch trainers in motivational interviewing), using the validated Motivational Interviewing Treatment Integrity (MITI) [18]. The MITI is a behavioural coding system assessing motivational interviewing skills. The MITI exists of two components: the global score and behaviour coding. The global score exists of five categories which are scored between one and five. Behaviour coding consists of a percentage of open and closed questions, a percentage of reflection (simple or complex), and a number of given information (motivational interviewing adherent or motivational interviewing non-adherent). CVRM knowledge was assessed in a questionnaire with six questions covering knowledge of lifestyle advice, CVRM treatment and risk factors. For practice nurses in the intervention group, the questionnaire was expanded, covering all components of the intervention programme. These included motivational interviewing, web programme CVRM, information cards, E-health, Twitter consultations, physical exercise group and referral to the GP, practice nurse mental health or psychologist. Practice nurses were asked which components they had used, whereby three answer categories (yes/partly/no) were provided. All patients received a composite questionnaire after the intervention period with questions on referral as part of the intervention programme and whether they used this advice. Answer categories were yes/no/once/several times, as applicable to the question concerned. The PHQ9 questionnaire was also included, which comprised of a 0–3 Likert-type scale (not at all/several days/more than half of the days/almost every day) [16], to check whether patients without/with mild/with severe depressive symptoms were referred to the suited recommendations as we suggested.

#### Qualitative measures

All practice nurses in the intervention group were invited to participate in a face-to-face semi-structured interview at follow-up, to answer the question why practice nurses used or did not use our intervention programme. Questions covered all components of the intervention programme. After four and 12 interviews with practice nurses, interim analyses were performed wherein the interview script was modified and questions were added about how practice nurses coped with possible depressive symptoms, high blood pressure or high cholesterol levels. During the semi-structured interviews, practice nurses were asked whether they felt that their skills to guide and motivate patients had improved after having attended the suggested trainings. Telephone interviews were held with patients to examine how they perceived the various components of the intervention programme. We asked patients whether they received a referral, whether they acted upon that advice and what their experiences were about the advice. Of four general practices, 29 patients who visited their general practice during the intervention period were invited by using convenience sampling. We invited only patients who visited the general practice; in doing so, we were able to generate more specific information about our intervention programme. Four research assistants working on the TICD project conducted the interviews with both practice nurses and patients. All interviews were audio-taped and transcribed verbatim.

### Data analysis

For quantitative analysis, we used SPSS (version 20, IBM Corp.) and for qualitative analysis, we used Atlas.ti7.

#### Quantitative analysis

In a multi-level regression model, the MITI scores were compared between baseline and follow-up, and between intervention group and control group. A two level model was used: measurements nested within practice nurses and practice nurses nested in general practices. Furthermore, we added time of measurement (baseline and follow up), allocation to research group (intervention group and control group) and the interaction term between time of measurement and allocated research group to the model. Other independent variables included were the global score and behaviour coding. Following the MITI code, the global score should be above 3.5; it is deemed desirable that the percentage of open questions is above 35%, and complex reflection above 40%; and only two times non-adherent information should be given such as advising without permission, making suggestions, or using words such as should, consider and try. A significant difference was set at *p* < 0.05. To assess knowledge about CVRM, questions were formulated on a basis of casuistry. All answers were checked using the answer key, whereby missing values were scored as wrong answers providing a conservative picture about the knowledge of practice nurses. Descriptive statistics were used to measure both the correct and wrong answers for the intervention group as well as the control group. An independent sample *t* test was used to measure the difference in correct answers between the intervention group and the control group. Results of the practice nurses’ questionnaires were used to measure which components of the intervention programme were used. Descriptive frequencies were calculated for each intervention element. Results of patients’ questionnaires were analysed by using descriptive crosstabs. First, we measured whether patients were referred by practice nurses to some components of the intervention programme, and second, we measured whether patients acted upon this advice. The answer categories whether patients acted upon this advice once or several times were merged for better data processing. We checked whether patients visited the general practice during the intervention period, whether they had been exposed to the intervention programme, whether they perceived no, mild, or severe depressive symptoms, and lastly, whether patients were referred to the suited option regarding depressive symptoms. Results of the PHQ9 questionnaires are normally divided into scores of 5, 10, 15 and 20, and represent cut points for mild, moderate, moderately severe and severe depression, respectively [16]. For this study, we considered a score up to 5 as no depressive symptoms, 6 to 15 as mild depressive symptoms and a score above 15 as severe depressive symptoms.

#### Qualitative analysis

The transcribed interviews were analysed using a two-stage content analysis [19]. The first three interviews of practice nurses and of patients were coded independently by two researchers. These results were discussed, and agreements were made for further coding. The remainder of the interviews were coded by one researcher and checked by a second researcher. Discrepancies were resolved through discussion. First, open coding was applied by coding all quotes relating to the tailored intervention programme. All quotes were transferred into an Excel data file. Then, axial coding was applied, whereby quotes were clustered per element of the intervention programme. The following coding scheme was used: motivational interviewing, web programme CVRM, information cards, E-health, Twitter consultations, physical exercise group and referral of patients with severe depressive symptoms. Furthermore, selective coding was applied by summarising important subthemes of the quotes mentioned. Axial and selective coding were performed by two researchers and checked independently by two other researchers. Consensus was reached through discussion. After this initial stage of content analysis, we categorised determinants of practice into domains of the TICD framework [14] in a second stage. We will present the results following the TICD framework which consists of the following domains: guideline factors, individual health professional factors, patient factors, professional interactions, incentives and resources, capacity for organisational change, and social, political and legal factors. We classified all determinants mentioned by practice nurses and patients during the process evaluation about the intervention programme where possible according to the seven domains, including the results of the feedback training motivational interviewing and the web programme CVRM. Some mentioned quotes associated with the intervention programme could not be classified by the framework. The second stage of content analysis was performed by one researcher and checked by two other researchers.

## Results

A total of 34 practice nurses started with the intervention programme. General practices were randomly allocated to the intervention group (19 practices, 20 practice nurses; two general practices with two practice nurses each and one practice nurse who worked in two participating general practices) and the control group (15 practices, 14 practice nurses; one practice nurse who worked in two participating general practices), see Table 1. One practice nurse in the intervention group dropped out just before the end of the intervention period due to a change of job and was therefore not available for this evaluation. Two audio tapes for the MITI were handed in by each of the 30 practice nurses, while four nurses handed in one recorded consultation at the start of the programme; all recorded consultations were used for analysis. All 19 practice nurses in the intervention group were interviewed, two men and 17 women; all interviews were held face-to-face except for one interview, which was conducted by telephone. The interviews lasted on average 36 min (range 12 to 67 min).

**Table 1:** General practice, practice nurse and patient characteristics

	Intervention group	Control group
Practice characteristics	*N* = 19	*N* = 15
Single-handed practice Duo/group practice	*N* = 10 (52.6%) *N* = 9 (47.4%)	*N* = 9 (60%) *N* = 6 (40%)
Rural area Urban area	*N* = 10 (52.6%) *N* = 9 (47.4%)	*N* = 6 (40%) *N* = 9 (60%)
Mean number of patients visiting the general practice per week (SD)	185.31 (91.56)	141.00 (48.34)
Mean FTE practice nurses (SD)	0.71 (0.35)	0.65 (0.32)
Practice nurse characteristics	*N* = 20	*N* = 14
Sex % female	90	100
Mean age in years	42	43
Mean number of years experience as practice nurse	12	11
Mean number of hours previous training of motivational interviewing skills	15	15
Patients characteristics	*N* = 1250	*N* = 979
Sex % female	35.1	34.9
Mean age in years (SD)	72.6 (9.2)	71.6 (9.7)
Number of patients that had a contact with the practice during the intervention period	*N* = 303	*N* = 161
Patient with established CVD	*N* = 130	*N* = 66
Patients at high risk	*N* = 173	*N* = 95
No symptoms of depression (%)	74.4	75.6
Mild symptoms of depression (%)	23.6	22.7
Severe symptoms of depression (%)	2.0	1.7

*Abbreviations*: *FTE* fulltimerequivalent

In total 1496 patients filled out the questionnaire, whereof 465 patients contacted the general practice during the intervention period, 303 patients in the intervention group and 161 patients in the control group. For the analysis, we only used information of patients in the intervention group who contacted the general practice, as only these patients could inform us on how our intervention programme was perceived. Twelve patients were interviewed, six women and six men; the telephone interviews lasted on average 23 min (range 12 to 29 min).

Results are presented in relation to the research questions. Results corresponding to research question 2 were classified to the TICD framework.

### Impact on counselling skills and CVRM knowledge (research question 1)

All practice nurses except one attended the feedback training motivational interviewing, which consisted of feedback directly after two patient contacts (one practice nurse only had one feedback moment due to lack of time). The MITI results showed small improvements, see Table 2. Not all parts of the behaviour coding of the MITI could be scored for all practice nurses because the audio-taped consults were too short, which explains the inequality of the number of practice nurses. The mean global scores for the intervention group were enhanced from 2.1 to 2.4 (scale of 1–5), the global scores in the control group decreased from 2.3 to 2.2. No significant difference (*p* = 0.169) was found between groups, after controlling for baseline scores. According to the MITI, the global score, which should be above 3.5, was only achieved by one practice nurse in the control group at the start (4.0), while another practice nurse in the intervention group achieved a global score of 3.6 at follow-up. The percentage of asking more open questions showed a significant difference between the intervention and the control group at follow-up (*p* = 0.009), although the overall score was below 35%. Furthermore, the score for complex reflections was not above 40% after the training, and the score for non-adherent information giving was far above the advised maximum of two times per consultation. Eleven practice nurses completed the web programme CVRM (including the practice nurse who dropped out later), four practice nurses started the web programme but did not complete it, while five practice nurses never started at all. On a scale from 0 to 6 correct answers on the knowledge questionnaire, the mean score of the intervention group was 3.4 correct answers in comparison with the control group who scored 4.5, a significant difference between groups (*p* = 0.048).

**Table 2:** Motivational interviewing skills (assessed with the MITI) comparing the intervention group and control group

		Intervention group at baseline	Intervention group at follow-up	Control group at baseline	Control group at follow-up	*P* value
Global scores (scale 1–5) (Should be above 3.5)		2.1 (*n* = 17)	2.4 (*n* = 17)	2.3 (*n* = 14)	2.2 (*n* = 13)	0.169
Behavioural coding (percentage open questions) (Should be above 35%)		17.3 (*n* = 17)	20.5 (*n* = 17)	25.0 (*n* = 14)	13.4 (*n* = 13)	0.009*
Reflection (percentage complex reflections) (Should be above 40%)		60.5 (*n* = 14)	27.1 (*n* = 17)	56.9 (*n* = 11)	9.1 (*n* = 12)	0.374
Information given	MI adherent (number of scored comments)	6.5 (*n* = 16)	5.7 (*n* = 15)	5.8 (*n* = 14)	7.9 (*n* = 13)	0.467
	MI non-adherent (number of scored comments) (Should be twice maximum)	6.3 (*n* = 16)	7.7 (*n* = 17)	5.6 (*n* = 14)	9.4 (*n* = 13)	0.474

Numbers between brackets = number of records which were scored by the trainers

*Abbreviations*: *MITI* Motivational Interviewing Treatment Integrity

*p < 0.05 = difference between intervention group and control group, controlled for baseline scores

### Use of interventions by practice nurses (research question 2)

Table 3 provides figures on the use of interventions resulting from the practice nurses’ questionnaire, and Table 4 provides figures on how many patients were referred to the components of the intervention programme. The questionnaire was filled out by 29 practice nurses, 16 practice nurses in the intervention group and 13 practice nurses in the control group. The uptake of the different components of the intervention programme ranging from 6.25 to 75.00% (Did you use the PHQ-9 questionnaire?). These figures will be clarified with reasons mentioned by practice nurses during the interviews, categorised by TICD domains. Most determinants mentioned by practice nurses and patients belong to two domains: individual health professional factors and capacity for organisational change. Practice nurses mentioned no determinants in two domains: guideline factors and social, political and legal factors.

**Table 3:** Use of interventions by practice nurses (*n* = 16)

	Yes	Sometimes	No	Missing
Did you pay attention to possible depressive symptoms in patients with established CVD or at high risk?	8 (50.00%)	5 (31.25%)	3 (18.75%)	0 (0%)
Did you use the PHQ-9 questionnaire?	3 (18.75%)	1 (6.25%)	12 (75.00%)	0 (0%)
Did you refer patients without depressive symptoms to the two websites?	2 (12.50%)	6 (37.50%)	7 (43.75%)	1 (6.25%)
Did you adopt the recommendation to refer patients without depressive symptoms to the two websites?	4 (25.00%)	4 (25.00%)	5 (31.25%)	3 (18.75%)
Did the recommendation to refer patients to the two websites enhance patients’ self-management?	1 (6.25%)	7 (43.75%)	5 (31.25%)	3 (18.75%)
Did you give patients the information card?	1 (6.25%)	10 (62.50%)	4 (25.00%)	1 (6.25%)
Did you adopt the recommendation to give patients the information card?	4 (25.00%)	4 (25.00%)	5 (31.25%)	3 (18.75%)
Did you refer patients to the Twitter consultations?	1 (6.25%)	7 (43.75%)	6 (37.50%)	2 (12.50%)
Did you adopt the recommendation to refer patients to the Twitter consultations?	1 (6.25%)	2 (12.50%)	7 (43.75%)	6 (37.50%)
Did you refer patients with mild depressive symptoms to an exercise group?	3 (18.75%)	7 (43.75%)	5 (31.25%)	1 (6.25%)
Did you adopt the recommendation to refer patients to an exercise group?	2 (12.50%)	2 (12.50%)	8 (50.00%)	4 (25.00%)
Did you refer patients with severe depressive symptoms to the GP, practice nurse mental health or psychologist?	8 (50.00%)	0 (0%)	4 (25.00%)	4 (25.00%)
Did you adopt the recommendation to refer patients to the GP, practice nurse mental health or psychologist?	1 (6.25%)	1 (6.25%)	8 (50.00)%	6 (37.50%)

**Table 4:** Patients’ reports on exposure to components of the intervention (*n* = 303)

	Yes	No	Missing	Total
Did you receive an information card?	69 (22.8%)	212 (69.9%)	22 (7.3%)	303 (100%)
Did you preserve the information card?*	51 (73.9%)	17 (24.6%)	1 (1.5%)	69 (100%)
Did the practice nurse refer you to the two websites?	28 (9.2%)	239 (78.9%)	36 (11.9%)	303 (100%)
Did you view the websites?*	15 (53.6%)	10 (35.7%)	3 (10.7%)	28 (100%)
Did you found the websites useful?*	14 (93.3%)	1 (6.7%)	0 (0%)	15 (100%)
Did the practice nurse refer you to the twitter consults?	3 (1%)	228 (75.2%)	72 (23.8%)	303 (100%)
Did the practice nurse refer you to an exercise group?	17 (5.6%)	233 (76.9%)	53 (17.5%)	303 (100%)
Did you visit the exercise group?*	11 (64.7%)	2 (11.8%)	4 (23.5%)	17 (100%)
Did the practice nurse refer you to the GP, practice nurse mental health or psychologist due to possible depressive symptoms?	12 (4.0%)	233 (76.9%)	58 (19.1%)	303 (100%)

Data on follow-up questions relate to patients who responded affirmative on the main question

### Domain individual health professional factors

Practice nurses in the intervention group who paid attention to the recommendation to consider depressive symptoms and refer patients to the suited recommendations did this because they found it relevant. Reasons for not paying attention to these recommendations were the lack of knowledge of the linkage between depression and cardiovascular diseases and practice nurses felt that they were not the right person to diagnose and register this. Most practice nurses forgot to document these data in patients’ medical files. One practice nurse decided to place the supportive PHQ9 questionnaire in the waiting room so that patients could fill it out, although she did not use the results of the questionnaire. The information card was perceived as being of little value. Practice nurses had written down the target values elsewhere instead of on the information card or given it verbally. Practice nurses referred patients in particular to the website thuisarts.nl, which was known by most practice nurses and perceived as reliable. Some practice nurses did not consider these websites as helpful for CVRM patients. Reasons for not referring patients to Twitter consultations were this recommendation was perceived as not useful and practice nurses were not familiar with Twitter themselves. Patients were referred to an exercise group regardless whether they experienced mild depressive symptoms, simply because practice nurses found it difficult to recognise mild depressive symptoms in patients. Practice nurses who referred patients to an exercise group did this already before the intervention period; they already knew physical exercise groups in the area and were also familiar with the television programme ‘Nederland in beweging’. Practice nurses who did not refer patients mentioned that they did not have any patients with mild depressive symptoms. Most patients with severe depressive symptoms were already receiving treatment, so a few patients were eligible for this recommendation. As a result of this implementation programme, some practice nurses worked more consciously with patients with established CVD or at high cardiovascular risk: they recognised depressive symptoms more often. Practice nurses mentioned that their guiding and motivating skills had improved due to the feedback training and the web programme CVRM.

### Domain patient factors

Patients were deemed to be too aged for Twitter consultations and for that reason were not referred to this type of consultation.

### Domain professional interactions

Our recommendation for patients with severe depressive symptoms was not to give them lifestyle advice but to start with reducing depressive symptoms. Even so, practice nurses gave lifestyle advice anyway because this was agreed upon within their general practice.

### Domain incentives and recourses

Reasons for not paying attention to the recommendation to consider depressive symptoms and refer patients to the suited recommendations were lack of time and changes in the electronic medical record systems.

### Domain capacity for organisational change

Not all practice nurses saw CVRM patients during the intervention period, and CVRM was not very well organised in some general practices. Moreover, changes in personnel and other projects within the general practice led to the fact that our intervention programme was not as well used as intended.

### Experiences with the implementation programme

In general, most practice nurses mentioned that they successfully adopted components of the implementation programme and would continue to do so. Nevertheless, the intervention programme was perceived as too much. There were too many items to think about. Another bottleneck was the lack of follow-up support and reminders from the research team. For some practice nurses, it was not clear how their activities regarding the intervention programme could have been noted. Finally, the intervention programme became blurred and forgotten about, due to part-time jobs.

### Referral of patients and patient reported healthcare (research question 3)

Table 4 provides figures of patients’ reports on exposure to components of the intervention programme, ranging from 1% (Did the practice nurse refer you to the Twitter consultations?) to 93.3% (Did you find the websites useful?). Figures of patients who mentioned that they had been exposed to components of the intervention programme were restricted to those who contacted the general practice during the intervention period. Not all patients had been referred to the recommended treatment or support options considering their level of depressive symptoms.

Reasons for not visiting the recommended websites by patients were that some patients had no access to the Internet, while some patients never used the Internet when searching for health-related information. Most patients mentioned that they did not know what Twitter was. Half of the patients mentioned that they did not receive lifestyle advice about food, exercise or smoking cessation. None of the patients interviewed visited an exercise group, while none were referred to the GP, practice nurse mental health or physiologist related to depressive symptoms.

## Discussion

Although the intervention programme was tailored to predefined key determinants of practice and introduced to practice nurses according to a standardised script, on average half of the practice nurses used and adopted components of the intervention programme. The information card and the recommendation to refer patients to the website ‘thuisarts.nl’ were the elements in our programme mostly used by the practice nurses. Practice nurses did not distinguish between patients with and without depressive symptoms, although this was an important aspect of the intervention programme. Important reasons for the lack of adherence to the programme were that for some practice nurses, it was not clear how to note their activities in patients’ medical records regarding the intervention programme and therefore only a few practice nurses made records, lack of time and follow-up support. Some patients mentioned that they had been referred to some components of the intervention programme, while a small group of these patients actually used the interventions offered. These findings provide potential explanations for the absence of impact on evaluation outcomes of the cluster randomised trial.

In a previous stage of our research, targets for improvement were determined, determinants of practice were selected and suggestions for interventions were collected for our tailored intervention programme to address several determinants [10], see Fig. 1. The evaluation of the tailored programme was the last phase of this project [8], which showed that there was no improvement on the targets for improvement. Determinants of practice were still perceived to be relevant, what indicates that these determinants were well selected. Although the intervention programme addressed some of the determinants, healthcare professionals’ behaviour did not change. The selected elements of the tailored intervention programme did not meet all the expectations of the practice nurses, which may explain that not all elements were used as recommended. Also, the lack of reminders and follow-up support of the research team plays an important role in the failure of the use and implementation of the intervention programme.

This process evaluation showed that the determinant ‘motivational interviewing’ was influenced by the feedback training with exposure to all practice nurses. The motivational interviewing skills are relatively poor, despite interest of nurses and training received. Determinants such as ‘giving good advice to patients’ and ‘more attention to patients’ motivation’ were influenced by the feedback training motivational interviewing and the educational web programme CVRM. Practice nurses mentioned they were better able to guide and motivate patients after both training sessions. Although practice nurses are positive about their functioning, knowledge about CVRM remains suboptimal, including knowledge about the relation between depressive symptoms and CVRM. Practice nurses were positive about referring patients to the E-health option thuisarts.nl. These findings imply that the assumed logical model of change may be valid, but the intensity of the intervention programme should have been higher to have any impact.

Despite the fact that we have identified determinants of practice before developing an intervention programme to change healthcare professionals’ behaviour, this process evaluation revealed hardly any change in healthcare professionals’ behaviour [12]. Several process evaluations of randomised trials in which behaviour change was accomplished or wherein determinants were identified have been published. One study confirmed that it is difficult to change healthcare professional behaviours [20], despite the fact that the participants expressed initial enthusiasm for the intervention programme. It may be possible that our intervention programme needed more instruction tools, such as booklets or online tools. That way, practice nurses in the intervention group could constitute a network to inform and motivate each other. Practice nurses in the intervention group felt that they were better able to motivate and guide patients after the intervention programme, which incidentally was also seen in another study of TICD series. In that particular study, practice nurses also felt more confident in treating patients after participating in a tailored intervention programme, although no improvements in guideline adherence were found [21]. In our study, the implementation of the programme by practice nurses was disappointing: only half of them used and adopted only parts of the programme. Other research showed a considerable variation of adoption of interventions by practice nurses whereof one study showed better adoption of interventions than our study [22–24]. These intervention programmes were deemed important by practice nurses and they expected better patient results afterwards, yet time was found to be a restriction. Moreover, digital interventions were difficult, a fact that corresponds with our outcomes.

Knowledge and awareness of practice nurses about the correlation of CVD and depressive symptoms might be enhanced in initial training and continuing education of nurses. But what is also needed is more recognition among practice nurses of the role of mental health problems among practice nurses, even if they primarily care for patients with somatic chronic conditions. The practice nurse mental health can provide added value to healthcare professionals, such as treating CVRM patients experiencing depressive symptoms or providing the staff with general information and tools for recognition to provide these patients with adequate care [25]. Training on practice nurses’ motivational interviewing skills is also needed to enhance these skills or at least to prevent deterioration. Although healthcare professionals expressed a strong preference for more training on motivational interviewing skills, it seems unlikely that this can enhance motivational skills in preventive care for vascular conditions and diabetes [26–28]. This process evaluation revealed that determinants targeted by the intervention programme did not lead to improvement: motivational interviewing skills and knowledge of CVRM were not positively influenced and with that, the basis for behavioural change seems to be lost. Questions hereby are the following were the selected determinants indeed important and changeable in current healthcare? And were the chosen strategies feasible and could they have an important impact? Although the use and adoption of recommended practices by practice nurses were limited in this study, a systematic review found that on average tailored interventions improved professional practice [7]. Nevertheless, more in-depth research is needed on how a tailored intervention programme works. What is the best way to obtain and select determinants and strategies so that the best strategies can be used for a tailored intervention programme?

### Strengths and limitations

We used multiple angles to illuminate all components of the tailored intervention programme and in doing so, achieved a broader view of all aspects, which is a strength of this process evaluation. Although almost all practice nurses handed in the questionnaires and the requested audio tapes, and participated in the interviews, the evaluation was based on a small number of practice nurses, so quantitative results should be interpreted carefully. All practice nurses handed in the first audio tape and we had three missing at follow-up, although some audio tapes were handed in too late, which could fade the effect. We recruited patients by convenience sampling in only four general practices. For that reason, it is possible that we have missed information about patients’ perceptions of our intervention programme. Some patients indicated that they had not been referred to the websites, yet they did have an opinion about it. It could be that patients had already checked these websites, which provide health information. Therefore, the results of patients’ questionnaires need to be interpreted carefully.

## Conclusions

Half of the practice nurses only partly applied and adopted the interventions from the tailored implementation programme, because the intervention programme was perceived as too much, practice nurses perceived a lack of reminders and follow-up support from the research team and practice nurses recorded only a few referrals. The programme aimed at pre-defined determinants such as motivational interviewing skills, CVRM knowledge and self-management promotion using E-health, but scores on these determinants did not change. These findings probably explain the absence of outcomes found in the trial. The assumed logical model of change could still be valid, but apparently, practice nurses missed support from the project team for a better adoption.

## Abbreviations

CVD: Cardiovascular disease
CVRM: Cardiovascular risk management
GP: General practitioner
ICPC: International Classification of Primary Care
LDL: Low-density lipoprotein
MITI: Motivational Interviewing Treatment Integrity
PHQ-9: Patients’ Health Questionnaire
SBP: Systolic blood pressure
TICD: Tailored interventions for chronic diseases
